# Physical and Dosimetric Aspects of the Iridium-Knife

**DOI:** 10.3389/fonc.2021.728452

**Published:** 2021-11-09

**Authors:** Yiannis Roussakis, Georgios Anagnostopoulos

**Affiliations:** Department of Medical Physics, German Oncology Center, Limassol, Cyprus

**Keywords:** iridium knife, brachytherapy, dosimetry, high-dose-rate (HDR) brachytherapy, stereotactic ablative radiotherapy (SABR), stereotactic body radiation therapy (SBRT), stereotactic radiosurgery (SRS), iridium-192

## Abstract

The three-dimensional iridium-192 (^192^Ir) high-dose-rate (HDR) brachytherapy manifests itself as a high-precision, hypofractionated, dose-escalating, minimally invasive method in the armamentarium of contemporary radiation oncology clinical applications. In this study, the physical aspects of the ^192^Ir radionuclide are presented. Its dosimetric application in HDR brachytherapy for different anatomical sites (prostate, gynecological malignancies, liver, and intrathoracic tumors) as well as the corresponding dosimetric comparison with the stereotactic body radiation therapy (SBRT) techniques based on a representative selection of dosimetric publications is reviewed and illustrated.

## Introduction

Iridium-192 (^192^Ir) has been extensively used for high-dose-rate (HDR) brachytherapy (BT) for more than four decades in the remote afterloading BT systems. Its physical properties are presented in **Table 1** (1–5). Its medium energy range of X- and gamma-rays (mean energy: 370 keV) in its energy spectrum and its beneficial reference air kerma rate (RAKR) of 0.1091 μGym^2^h^-1^MBq^-1^ in comparison with other HDR source radionuclides such as ^60^Co (energy: 1.173 and 1.332 MeV, RAKR: 0.308 μGym^2^h^-1^MBq^-1^) have always required definitively less shielding than any external beam radiation modality. The tenth value layer (TVL) for ^192^Ir is 16 and 152 mm for lead and concrete, respectively, while for ^60^Co, the TVL is 41 and 218 mm for lead and concrete, respectively (4). This enables the construction of ^192^Ir HDR BT treatment rooms in a more cost-effective way and storage of the source inside the remote afterloader an easier task. The half-life of ^192^Ir is 73.81 days, enabling a relatively economic application of this radionuclide in remote afterloading HDR systems for radiation therapy, by implementing four source exchanges per year, in order to maintain the HDR of greater or equal to 12 Gy/h. Its main advantage is especially the high specific activity (340 GBqmg^-1^), which makes feasible the manufacturing and the distribution of high-activity ^192^Ir miniaturized size sources (370–480 GBq). The typical length of an encapsulated ^192^Ir HDR BT source is in the order of 5–11 mm, and the typical outer diameter lies in the order of 0.8–1 mm. For distances from the source center, r, greater than the double of the ^192^Ir active core length (r ≥ 2L ≈ 8 mm), the source can be considered a point source in its dosimetry (2, 3), and the dose rate distribution in the periphery of the source—hence the dose rate in the planning target volume (PTV) and in the surrounding healthy tissue—is determined by the inverse square law (r^-2^). The immediate contact of the implanted catheter/applicator inside the PTV, where the miniaturized ^192^Ir source is remotely driven, along with the predominance of the inverse square law of a single source in its dwell position, facilitates the characteristic steep dose fall-off, which in comparison with a single megavoltage external X-ray or even a proton beam (with the characteristic Bragg peak) is unbeatable.

**Table 1:** Physical characteristics of ^192^Ir radionuclide and ^192^Ir sources used in brachytherapy (1–4).

^192^Ir	
Half-life (days)	73.81
Type of disintegration	β^-^ (95.1%), Electron Capture (4.9%)
Maximum x-ray energy [keV]	78.6
Gamma energy range [keV]	110.4–1,378.2
Mean gamma ray energy [keV]	370
Air kerma rate constant, Γ_δ_ [μGy m^2^ h^-1^ MBq^-1^]	0.1091
Specific activity [GBq mg^-1^]	341.0
Range of active ^192^Ir source core length [mm]	3.5–10.0
Range of active ^192^Ir source core diameter [mm]	0.34–0.70
Tenth value layer (TVL) in lead [mm]	16
Tenth value layer (TVL) in concrete [mm]	152

d, days; keV, kilo electron-volt; μGy, micro-Gray; MBq, mega-Becquerel; GBq, giga- Becquerel; mm, millimeter; mg, milligram.

The characteristic dosimetric property of the ^192^Ir BT source with its sharp dose gradient justifies reasonably the name “Iridium-Knife” in correspondence to the high-precision, steep-dose gradient Linear Accelerator (LINAC)-based multi-leaf collimator and the robotic radiosurgery device-based external-beam radiotherapy, which are also known as X-knife (6) and Cyberknife (7), respectively.

The purpose of this mini-review is to illustrate the capability of the three-dimensional (3D) HDR BT with ^192^Ir for the delivery of conformal dose coverage of the PTV by concomitant dose escalation inside them and the steep dose fall-off in their periphery, acting thus as another precision dose knife, due to its physical properties. Furthermore, by reviewing representative published data for four different anatomical sites, a dosimetric comparison with the stereotactic body radiation therapy (SBRT) method is presented and the advantages as well as disadvantages of the ^192^Ir HDR BT are highlighted.

## Materials and Methods

In this article, we review dosimetric studies for prostate, liver, endometrium, and intrathoracic malignancies either clinically performed with HDR BT and afterward virtually planned with SBRT or *vice versa* (8–15). The dosimetric comparison for PTV coverage, organs at risk (OARs) dose sparing, plan conformity, dose heterogeneity, dose fall-off sharpness, and low dose spill are presented with the aid of dose-volume and physical quantities presented in **Table 2**. The clinical evaluation and the medical follow-up of the patients subjected to these radiotherapy modalities are beyond the scope of this article.

**Table 2:** Dose-volume and physical quantities used for the dosimetric assessment of the radiation therapy modalities.

Dose-volume parameters and other plan quality factors	Definition or equation describing the quantity	Commonly used dose volume quantities
**PTV**		
VX%	The volume of the PTV receiving X% of the prescribed dose (PD)	V100%, V95%, V90%, V150%, V200%, V105%
VXGy	The volume of the PTV receiving X Gy of dose	
DA%	The dose received by the A% of the PTV	D95%, D99%, D100%
D_min_	The minimum dose within the PTV as percentage of the PD	
D_max_	The maximum dose within the PTV as percentage of the PD (the same applies for the volume of the OARs)	
Conformity Index (CI)	V_PTV PD_/V_PTV_ (V_PTV PD_: volume of the PTV covered by the PD and V_PTV_: volume of PTV)	CI values close to 1.0 indicate better conformity of the PD to the PTV
Conformation number (CN)	(V_PTV PD_)^2^/V_PTV_ x V _PD_ (V_PTV PD_: volume of the PTV covered by the PD and V_PTV_: volume of PTV, V_PD_: volume of the prescribed dose)	
**OARs**		
VY%	The volume of the OAR receiving Y% of the prescribed dose (PD)	V20%, V40%, V60%, V80%, V100%
VYGy	The volume of the OAR receiving Y Gy of dose	
DB%	The dose received by the B% of the OAR volume	D10%, D20%
D_yccm_	The dose received by y cubic centimeters of the OARs	D_0.1ccm_, D_0.5ccm_, D_1ccm_, D_2ccm_, D_5ccm_, D_10ccm_, D_1,000ccm_
D_mean_	Mean dose received by the OAR	
Healthy Tissue Conformity Index (HTCI)	(V_PTV PD_)^2^/V_PTV_ x V _PD_ (it takes into account the irradiation of healthy tissue beyond the PTV)	
**Conformality and dose fall-off gradient indices**		
Conformal Index (COIN)	CN x $\Pi_{i}^{N}(1-\frac{V_{i,PD}}{V_{i}})$ (CN: conformation number, N is the number of OARs under consideration, *V_i,PD_* is the volume of the i-th OAR covered by the PD and *V_i_* is the volume of the i-th OAR)	Each of the COIN component would be ideally equal to 1.0
High dose spillage (HDS)	$\frac{V_{105\%\;for\;(body-PTV)}}{V_{PTV}}$ (it is the ratio of the volume outside of the PTV receiving 105% of the PD to the volume of the PTV	Any higher dose than 105% of the PD should not be found outside the delineated PTV
R_50%_	The ratio of the volume receiving 50% of the PD to the volume of the PTV	
D_2cm_	Maximum relative dose 2.0 cm beyond the PTV in any direction as percentage of the PD	
V20%–V80%	Healthy tissue volume outside the PTV receiving between 80% and 20% of the PD expressed in cm^3^	

PTV, planning target volume; PD, prescribed dose; OAR, organ at risk; ccm, cubic centimeter.

## Review of Representative Dosimetric Studies

### Prostate

For high- and intermediate-risk prostate cancer patients, a small number of planning studies have been carried out for the comparison of the established prostate HDR BT with the SBRT modality (8–10). It should be mentioned that the SBRT in the prostate was initially termed “virtual HDR” because of the similar characteristics to HDR BT, namely, hypofractionation and high dose per fraction administration.

In the first study (8), the patients were treated with ^192^Ir HDR BT with transrectal ultrasound (TRUS)-guided plastic needle insertion with a dose of 4 × 9.5 Gy as monotherapy. Following the recommendations of the American Brachytherapy Society, the dose parameters applied in the optimization phase were for the urethra D_max_ less than 120% of the prescribed dose (PD), for rectum D_max_ less than 100% of the PD and D_2ccm_ less than 70% of the PD, for the bladder D_2ccm_ less than 75% of the PD, and for the PTV coverage V100% greater than 90%. Spratt et al. used two approaches to dosimetrically compare the HDR dosimetry with the virtual SBRT plans. The first was the “normal tissue-prioritized” plan, where the primary goal was to maintain the OAR constraints by simultaneously trying to maximize target coverage, and the second approach was the “PTV-prioritized” plan, where the coverage of the PTV was the primary goal by allowing the constraints for the OARS to be violated in an as minimal as possible extent.

The dosimetric comparison results of the “normal tissue-prioritized” plans are presented in **Table 3** [**Table 1** (8)] and reveal that all dose metrics for the rectum and the bladder were lower for the HDR BT, but only the rectal D_max_ reached statistical significance, representative of the sharper dose fall-off outside of the PTV for the Iridium-Knife. As expected, dose metrics for the urethra were always higher for the HDR BT in comparison with SBRT due to the predominant inhomogeneity of the dose distributions around the source dwell positions inside the PTV and in the proximity of the urethra, a characteristic that is evident with the dose escalation feature and the resulting value of V200% for the HDR BT inside the PTV, a value that cannot be reached by SBRT.

**Table 3:** The results of the dosimetric comparison of the normal tissue-prioritized plans from the study of Spratt et al. (8) [courtesy of Spratt et al. (8), reproduced with permission].

Dosimetric parameter	SBRT plans	± Standard Deviation	HDR plans	± Standard Deviation	*p*-value
PTV V100%	93.08%	3.20	93.78%	1.78	Non-significant
PTV V150%	42.86%	7.70	40.32%	6.47	Non-significant
PTV V200%	0.00%	0.00	15.18%	3.05	0.00
Rectum D_max_	99.42%	2.79	94.24%	5.24	0.05
Rectum D_2ccm_	71.14%	4.78	60.84%	5.90	0.07
Rectum D_mean_	28.43%	4.00	27.12%	4.03	Non-significant
Bladder D_max_	110.06%	9.92	104.17%	30.05	Non-significant
Bladder D_2ccm_	78.78%	6.41	58.30%	9.58	0.08
Urethra D_max_	115.80%	5.40	119.28%	3.98	Non-significant
Urethra D_1ccm_	75.17%	29.72	87.72%	12.87	Non-significant
Urethra D_mean_	84.83%	13.11	95.04%	9.96	0.08

PTV, planning target volume; VX%: volume receiving X% of the prescribed dose; Dmax, maximum dose within a given region; Dmean, mean dose within a given region; Dyccm, dose received by y cubic centimeters of a given region.

For the case of “PTV-prioritized” plans, where an attempt was made for SBRT to match the PTV V200% of the HDR BT by relaxing the OAR constraints, the PTV V150% was significantly higher (67%) for SBRT than for the HDR BT plan (40%) (*p* = 0.045). Furthermore, rectum D_max_ and D_mean_ for the SBRT were 111% and 33%, respectively, while in HDR BT, they were significantly lower 94% (*p* = 0.045) and 27% (*p* = 0.028), respectively, thus violating the dosimetric constraints for rectum. Additionally, the bladder and urethral doses were higher for SBRT in this approach compared to HDR BT, without showing statistical significance. Another important finding of this study is that, for this plan approach, the surrounding healthy tissue total body dose was significantly greater for SBRT than for HDR BT (V10% = 2,206 cm^3^ for SBRT vs. V10% = 1,250 cm^3^ for HDR BT, *p* = 0.01), implying that the dose escalation with SBRT inside the prostate PTV to approach HDR BT comes with a cost of increased dose spillage in the surrounding healthy tissue.

In the second study (9), six consecutive prostate patients have been treated HDR BT with a dose prescription of 45.5 Gy in seven fractions. Metallic needles were placed with real-time TRUS guidance, and the plan was created on a CT image acquired after needle insertion. The clinical target volume (CTV) comprised the prostate gland plus 5 mm in all directions to cover possible extracapsular extensions, except for the posterior rectal margin, which varied from 2 to 5 mm depending on the distance from the rectal wall. The planning goal in that study was to achieve a V100% of partially lower than 100% (because V100% = 100% is impossible due to the interference of the metallic needle positions with the urethra shape in the anterior ventral part of the prostate that cannot be intersected, thus leaving a cold spot in that area), with urethra D_max_ lower than 150% of the PD and rectum D_max_ lower than 100% of the PD.

The virtual SBRT (CyberKnife) planning was performed with the CT images and structures from the HDR BT plan, except for the PTV that was adjusted with an additional margin of 2 mm. The SBRT dosimetric constraints were for the PTV: dose within 100% and 150% of the PD, D_max_ for rectum, urethra, and bladder to be less or equal to 100% of the PD. For each SBRT plan, the most common prescription method of D95% was used as the prescription dose to perform the dosimetric comparison of SBRT with HDR BT.

The results for dose volume parameters of the PTV [**Table 1** (9)] revealed that, in terms of dose escalation inside the PTV, the HDR BT is significantly better than the SBRT modality. However, the D99%, the D100%, and the V100% SBRT values are higher than the corresponding ones of HDR BT due to the inherent limitation of the HDR BT to dosimetrically fully cover with the PD the anterior part of the PTV, where the urethra is located, because the urethra should not be penetrated with needles. Considering the dose received by lower volume fractions of the PTV, such as the D90%, HDR BT performed significantly better than SBRT. Moreover, the V125%, the V150%, and the V200% values of HDR BT are significantly higher than the ones of SBRT, and this dose escalation could lead to a better outcome (16).

The dosimetric comparison for the rectum revealed that HDR BT had a higher D_max_ and a steeper dose fall-off inside the rectum than SBRT in the intermediate to high dose range. The average D_1ccm_, D_2ccm_, D_5ccm_, D_10ccm_, and D_20ccm_ were significantly lower for HDR BT by 5.1, 7.1, 7.6, 6.1, and 2.4 Gy, respectively. Moreover, the average V40%–100% values were also significantly lower in HDR BT, even though they were relatively small (1.6–4.5 cm^3^). For the urethra, the HDR BT was inferior to SBRT with statistical significance. The average D_0.1ccm_, D_0.2ccm_, D_0.5ccm_, D10%, and D20% were considerably higher than for SBRT by 12.6, 8.9, 4.5, 10.2, and 7.0 Gy, respectively. For the bladder, HDR BT resulted in higher doses than the SBRT modality, with no statistical significance however, except for the D_0.5ccm_, which was equal to 96.2 Gy for HDR BT and 51.1 Gy for SBRT (*p* = 0.02). This might be attributed to the fact that, for the HDR BT, source dwell positions of catheters inside the bladder pouch were activated in order to be able to cover the anterior cranial part of the prostate PTV with the 100% of the PD, resulting in very high doses in the bladder.

In the third study (10), 15 patients were treated with SBRT (CyberKnife) with a dose prescription of 35 Gy in five fractions. TRUS-based fiducial marker implantation was performed at least 5 days before treatment planning CT acquisition, and a TRUS dataset was acquired immediately before implantation. For the SBRT plans, GTV was defined as the prostate on MRI in low risk and prostate with base of seminal vesicles (1 cm proximal) for all other patients. CTV was generated by uniform expansion of the CTV by 2 mm, while PTV was generated by anisotropic expansion of the CTV (1 mm posterior and 3 mm in all other directions). The planning goal was to achieve V100% of more than 95% for the PTV and V37.5Gy of more than 95% for the GTV. The dosimetric constraints for rectum D_max_ were lower than 38 Gy, and V36Gy, V29Gy, and V18Gy encompassing less than 1, 15, and 25 cm^3^, respectively. For bladder, the D_max_ was lower than 38 Gy, with V36Gy less than 10 cm^3^ and V18Gy covering less than 40% of its volume. For urethra, Dmax was lower than 44 Gy and V44Gy less than 20% of its volume.

The virtual HDR BT plans were created on the aforementioned TRUS images with a dose prescription of 35 Gy in a single fraction in order to allow direct dosimetric comparison to the clinical SBRT plan. The PTV encompassed the entire prostate gland without margins. The planning goal was to achieve D90% of more than 100% and D150% less than 35% for the PTV. The dosimetric constraints for rectum D_0.1ccm_ were less than 80% of PD for rectum and bladder and less than 120% of the PD for urethra.

The dosimetric comparison for the prostate (i.e., SBRT GTV or HDR BT PTV) showed that the dose delivered to 98% of the volume, the V35Gy and V37.5Gy were significantly higher for SBRT compared to HDR BT plans, while V42Gy and V52.5Gy were significantly higher for HDR BT compared to SBRT plans. The maximum dose to the rectum and bladder was significantly lower, while the maximum dose to the urethra was significantly higher in HDR BT plans compared to SBRT plans.

### Liver

A small number of studies thus far attempted to dosimetrically compare brachytherapy plans against SBRT plans in the treatment of primary or secondary malignancies of the liver (11, 12).

Pennington et al. (11) investigated the differences of brachytherapy and SBRT plans in a retrospective study of 10 patients with liver metastasis, originally treated with SBRT, and for which virtual ^192^Ir HDR BT plans were created. Both plans were designed to deliver five fractions of 12 Gy to the same PTV, while no precise information is given for the generation of this volume. The stated HDR BT planning goal was to match the PTV receiving 100% of the PD to the SBRT plan. In terms of target coverage, they found that the mean PTV V100% was comparable between HDR BT and SBRT (94.1% vs. 93.9%, *p* = 0.8), while mean PTV V150% was significantly higher in HDR BT plan (63.6% vs. 0%), revealing significant dose escalation. On the other hand, significantly lower minimum dose as a percentage of the prescription dose within the PTV was exposed for HDR BT compared to SBRT plans (66% vs. 88%, *p* = 0.0002). They found no statistically significant differences (*p* = 0.109) in dose fall-off as estimated by R50%. The authors concluded that HDR BT can achieve higher target dose, similar dose to OARs but potentially lower target coverage in comparison with SBRT (11). A limitation to this study is the retrospective virtual HDR BT planning of the data, coupled with the fact that the same PTV was irradiated as for the SBRT plans. This is typically not the case in clinical practice where the CTV is considered to be the PTV in HDR BT treatments, since due to the fact that needles are fairly stable within the tumor, the uncertainties addressed by the PTV expansion are deemed negligible. Therefore, even though it is a fact that in HDR BT clinical practice, it is often difficult for the prescription dose to cover the entirety of the PTV, a fairer comparison of the two modalities would compare the coverage of the prescription dose for the respective PTV employed for each modality.

In a recent study, Hass et al. (12) performed a similar comparison of HDR BT vs. SBRT plans for liver lesions while addressing the aforementioned limitations of previous studies. In their article, 85 patients previously treated for liver malignancies with HDR BT were used, and for each, a retrospective virtual SBRT plan was generated. The GTV was generated based on contrast-enhanced breath-hold CT scanning with a 3–5-mm margin for the generation of the CTV, depending on the visualization quality of the GTV. No additional margin was added for HDR BT planning, and therefore, CTV = PTV for this case. In contrast, a margin of 5 mm in lateral direction and 10 mm along the craniocaudal axis was added to the GTV for the generation of the SBRT-specific PTVs. The prescription dose for both treatments was the same for both plans (15 or 20 Gy in one fraction, depending on type of tumor) and was prescribed for both treatment modalities to be 99.9% of the PTV. The same OAR constraints were employed. The dosimetric results of this investigation are summarized in **Table 4**. They revealed significantly better coverage PTV D99.9% by the prescribed HDR BT plans compared to SBRT plans in both the 15 Gy (*p* < 0.001) and 20 Gy (*p* = 0.003) groups. Similarly, mean PTV D90% was significantly higher in the HDR BT plans in both the 15-Gy (*p* < 0.001) and 20 Gy (*p* < 0.001) groups. Regarding the exposure of the remaining liver volume, the study found no statistically significant differences in V5Gy in the 15 Gy group (*p* = 0.095), but statistical significance was found in the 20 Gy group (*p* = 0.001) in favor of the HDR BT plans. The authors concluded that HDR BT revealed superior outcomes both in terms of target coverage (PTV D99.9% and D90%) and exposure to the remaining healthy liver. They also discussed further advantages to each of the examined modalities. Namely, HDR BT allows for irradiation of larger liver tumors (SBRT is typically limited to sizes with diameter less than 4–6 cm) and more centrally located tumors (for which SBRT is typically avoided) while also being less affected by uncertainties related to respiratory breathing motion. On the other hand, HDR BT is a minimally invasive procedure while SBRT is non-invasive. The authors point out some limitations in their study. The main one is the fact that their planning procedure for SBRT was aiming for a relatively homogeneous dose distribution inside the target, avoiding central dose escalation. This would typically lead to a shallower dose gradient than would otherwise be achieved if the authors allowed the SBRT treatment planning optimizer the flexibility to have a higher dose in the central region of the PTV. Therefore, steeper dose fall-off would be expected with current SBRT planning strategies that allow up to 125%–140% of the prescription dose within the target.

**Table 4:** The dosimetric results (mean ± standard deviation) of the different brachytherapy (BT) and stereotactic body radiotherapy (SBRT) from the study of Hass et al. (12) [courtesy of Hass et al. (12), reproduced with permission].

	Overall			15-Gy prescription dose			20-Gy prescription dose		
	BT	SBRT	*p*	BT	SBRT	*p*	BT	SBRT	*p*
D_90%_ [Gy]	27.9 ± 0.4	19.5 ± 0.3	<0.001	24.3 ± 0.8	16.5 ± 0.3	<0.001	29.2 ± 0.4	20.6 ± 0.3	<0.001
D_99.9%_ [Gy]	18.8 ± 0.4	16.8 ± 0.4	<0.001	16.0 ± 0.4	14.7 ± 0.4	0.003	19.9 ± 0.4	17.5 ± 0.5	<0.001
Liver V_5Gy_ [cm^3^]	593 ± 36	671 ± 33	<0.001	544 ± 65	607 ± 71	0.098	611 ± 43	694 ± 43	0.674
Liver V_5Gy_ [%]	39.5 ± 2.0	43.6 ± 1.7	0.001	33.3 ± 2.7	37.3 ± 3.0	0.095	41.8 ± 2.5	45.9 ± 2.0	0.977

BT, brachytherapy; SBRT, stereotactic body radiotherapy; DA%, dose received by A% of a given region; VXGy, the volume of a given region receiving X Gy of dose.

### Gynecological Malignancies

A number of investigators performed retrospective studies to dosimetrically compare HDR BT and SBRT sequential boost in the treatment of gynecologic malignancies, and two representative investigations are discussed herein (13, 14).

Georg et al. (13) attempted a comparison of HDR BT against SBRT in cervical cancer using high-tech techniques for both methods. Nine patients with locally advanced cervical cancer that were previously treated with HDR BT boost under MRI guidance were employed. Additional SBRT plans were created with step-and-shoot intensity-modulated radiotherapy (IMRT) and intensity-modulated proton therapy (IMPT) for each patient. Both SBRT treatment plans were created under the assumptions that (a) MRI-guided beam delivery was available, (b) dedicated applicator for cervix immobilization would be used, (c) online adaptive planning would be employed, and (d) sufficient patient immobilization would exist. For HDR BT plans, the PTV was taken to be the same as the CTV, while for the SBRT plans, two PTV scenarios were created, with 5- and 3-mm expansion of the GTV for the generation of the SBRT-specific PTV. For all cases, the plans created to deliver four fractions of 7 Gy. HDR BT plans were prescribed such that the prescription dose would fully cover the PTV, while SBRT plans aimed to deliver the highest possible dose to the GTV and PTVs [intermediate-risk PTV (IR-PTV) and high-risk PTV (HR-PTV)] while maintaining the same OAR DVH parameters previously achieved by the HDR BT plans. Since the SBRT planning constraints adopted the HDR BT-achieved OAR doses, these were very similar. The authors revealed that for IMRT plans limited to the HDR BT-achieved OAR constraints, GTV or PTV D90% was in general lower than the respective HDR BT plans. For IMPT plans, D90% was mostly similar or lower to that of HDR BT plans. When ratios of volumes receiving 3 and 3.5 Gy were compared, IMRT plans revealed volumes twice as large, and volumes receiving 5 Gy were 1.5 times as large as HDR BT plans, regardless of the PTV margin size. Volumes receiving 7 Gy were on average smaller in IMRT than HDR BT plans. For IMPT, volumes receiving 3, 3.5, and 5 Gy were approximately 1.5 times larger compared to HDR BT plans. They concluded that for image-guided cervical cancer sequential boost treatments, both IMRT and IMPT seem to be inferior to HDR BT (13).

Yildirim et al. (14) recently performed a similar retrospective dosimetric study comparing HDR BT plans against SBRT plans designed with VMAT technique on a conventional linear accelerator (Axesse, Elekta AB, Stockholm, Sweden) and on a Hi-Art Tomotherapy system (TomoTherapy Inc., Madison, WI). Twelve patients with early-stage endometrial cancer previously treated postoperatively with HDR BT were included and for which SBRT plans were retrospectively created with the two abovementioned technologies. The PTV was defined as a uniform 3D 5-mm expansion of the cylinder volume at the upper 3–5 mm of the vagina and was the same for all planning cases. The PD was 25 Gy in five fractions. The goal of the plan in both HDR BT and SBRT cases was that 95% of the PTV to receive at least 95% of the PD and 100% of the target volume to receive at least 90% of the PD. The authors reported total coverage of target volumes with 150%–250% of the PD for the HDR BT plans. The mean PTV D98% was 24.71 ± 0.36 Gy for the TomoTherapy plans and 24.42 ± 0.38 Gy for the VMAT plans, i.e., slightly lower than the prescription dose and were deemed adequate. Regarding the OAR doses, bladder D_2cc_ was found significantly lower in HDR BT plans than in VMAT and TomoTherapy plans, with no significant differences observed in the rectum D_2cc_ between the three plans. The authors concluded that their investigation demonstrated comparable PTV coverage between SBRT and HDR BT plans with lower inhomogeneity in the SBRT compared to the HDR BT plans and claimed that this study showed the feasibility of using SBRT as an alternative to HDR BT in endometrial cancer patients treated postoperatively (14). A limitation to this study was the use of the same PTV in both SBRT and HDR BT plans, created with the expansion of the CTV, even though it is unlikely to be performed clinically due to the inherent positioning uncertainties associated with external beam radiotherapy treatments.

As a result of a growing recent utilization of SBRT treatments instead of the established HDR BT option in clinical settings, Gill et al. (17) utilized the United States National Cancer Database for the evaluation of the potential impact of SBRT usage in cervical cancer treatments. A total of 7,654 patients with stage IIB–IVA cervical cancer for which boost modality information was available were used. Of these patients, 90.3% received HDR BT and the rest received IMRT or SBRT. It was observed that from 2004 to 2011, the use of brachytherapy decreased from 96.7% to 86.1%, while use of IMRT and SBRT increased from 3.3% to 13.9% (*p* < 0.01). The comparative survival analysis between the two modalities revealed that IMRT or SBRT boost resulted in inferior overall survival (hazard ratio, 1.86; 95% confidence interval, 1.35–2.55; *p* = 0.01) when compared to HDR BT boost. The authors concluded that the increased use of IMRT and SBRT techniques for delivering the boost dose in cervical cancer patients with the concurrent apparent increase in mortality risk should raise concerns when deciding on the use of these modalities over HDR BT outside of clinical trials.

Addressing the concern raised by the analysis by Gill et al. (17), the Society of Gynecologic Oncology and the American Brachytherapy Society recently published a review article (18) that includes a discussion on the comparison of brachytherapy with IMRT or SBRT. They conclude with the statement “(…) conformal external beam therapies such as IMRT or SBRT should not be used as alternatives to brachytherapy in patients undergoing primary curative-intent radiation therapy for cervical cancer”.

### Intrathoracic Malignancies

In the study of Milickovic et al. (15), five patients with intrathoracic malignancies (IMs) of different sizes, that received X knife SBRT, were selected for ^192^Ir HDR BT comparative plan analysis. These patients were planned with the treatment planning system (TPS) Oncentra MasterPlan v4.5 (Elekta, Veenendaal, Netherlands) with 9–10 non-coplanar 6 MV X-ray beams delivering doses from 5 Gy up to 20 Gy per fraction. In order to ensure healthy tissue sparing, the planned dose encompassing the PTV was set equal to 80% of the isocenter dose. By using the same structures (PTV and OARs), they generated HDR BT virtual plans with the Oncentra Brachytherapy v4.5 TPS, with the planning goal to achieve the same PTV with the PD of the SBRT and the aid of the hybrid inverse optimization algorithm installed in the TPS (19). The number of virtual catheters for the BRT plans ranged from 6 to 10, and the number of activated dwell positions pro cm^3^ was 3. For the comparative dosimetric analysis, DVH was calculated and the paired Student’s *t*-test with a *p*-value of 0.05 as significance threshold was applied.

The results of this study show a significantly better PTV coverage (*p* = 0.030) with the HDR BT for the V100% (93.04% of the PD) vs. the SBRT (88.94% of the PTV), while the other VX% and DX% parameters revealed no significant benefit of the HDR BT over SBRT, although the values are always greater for the HDR BT in comparison to the corresponding SBRT ones, except for the D_min_. The value of V150% for HDR BT equals to 24.67, while for SBRT is 0.0, as expected, indicating the ability of HDR BT for significant dose escalation inside the PTV (*p* = 4.84 × 10^-4^).

The CI, HTCI, CN, and COIN parameters did not show significant differences. For the high-dose spillage, there was no significant difference shown for both treatment methods because the amount of healthy tissue receiving doses more than 105% of the PD is low. For the intermediate-dose spillage, the R_50%_ of the HDR BT treatment plans was significantly (*p* = 0.002) better (2.47) than the corresponding one of the SBRT (21.14), D_2cm_ was significantly higher for the SBRT treatment plans than for the HDR BT (*p* < 0.001), and V20%–V80% was significantly in favor of HDR BT (*p* = 0.003). In the same study, it has been illustrated that this volume difference favors the usage of HDR BT with increasing PTV. The comparison of the dose indices of the OARs exhibited no statistically significant difference of the two methods, except for the D_max_ in the spinal cord, where for HDR BT, the average value equals to 12.85Gy ± 7.42% and for the SBRT equals to 21.14Gy ± 5.72% of the PD (*p* = 0.022).

The study of Milickovic et al. (15) showed that SBRT is not dosimetrically superior to HDR BT and furthermore that HDR BT can deliver higher dose escalation inside the PTV in intrathoracic cases. Regarding larger lesions, where SBRT might not be able to deliver a sufficient dose due to normal tissue dose constraints, HDR BT is shown to manifest itself as a therapeutic option, considering the dosimetric aspect, although the results of the study might differ, if real implants are considered, because catheter placement alters the PTV anatomy.

### Cerebral Malignancies

Milickovic et al. (15) also selected five patients with cerebral malignancies (recurrent glioblastoma multiform) who received X knife stereotactic radiosurgery (SRS) for retrospective dosimetric comparison against ^192^Ir HDR BT. SRS and HDR BT plans were generated with the same considerations described above for the intrathoracic malignancies in the same publication.

The results show that similar coverage was achieved by the two modalities (HDR BT: 96.09%, SRS: 94.73%) with no statistically significant differences (*p* = 0.227). As mentioned above, VX% and DX% parameters revealed no statistically significant differences besides V150%, which was significantly higher for HDR BT compared to SRS plans.

The HTCI, CN, and COIN parameters did not show significant differences besides CI, which was significantly better (*p* = 0.026) for HDR BT than for SBRT. No significant differences were observed for R_50%_, while D_2cm_ and V20%–V80% revealed significant differences in favor of HDR BT (*p* = 0.032 and *p* = 0.035, respectively). Dose incidence for OARs did not reveal any significant differences either between the two treatment modalities.

Milickovic et al. (15) conclude that HDR BT is at least as good as SRS in cerebral malignancies—when the anatomical position of the target allows needle placement—the steep dose fall-off was shown to be sharper in HDR BT plans, while intermediate- and low-dose spillage was as good or significantly lower in HDR BT plans.

## Discussion and Conclusions

This article deals with the illustration of the basic ^192^Ir physical properties, which justify its steep dose fall-off in its application for the interventional radiation oncology. Additionally, it reviews representative dosimetric comparison studies of the SBRT vs. the HDR BT modality (summary in **Table 5**). The clinical comparison and analysis review are beyond the scope of this article, as it is also for the assessment of the impact of the uncertainties each of the two modalities is linked to.

**Table 5:** Summary of study year, original clinical plan, quantitative treatment plan analysis metrics employed, and number of patient cases utilized for the studies mentioned in current review.

	Study Year	Clinical Plan	Quantitative treatment plan analysis			No. of test cases
			PTV or CTV	OARs	Other	
**Prostate**						
Spratt et al. (6)	2013	HDR BT	V100%, V150%, V200%	D_max_, D1ccm, D2ccm, D_mean_	–	5
Fukuda et al. (7)	2014	HDR BT	V100%, V125%, V150%, D90%, D95%, D100%	V20%, V40%, V50%, V60%, V80%, V100%, D0.1ccm, D0.2ccm, D0.5ccm, D1ccm, D2ccm, D5ccm, D10ccm, D20ccm, D30ccm, D40ccm, D50ccm, D80ccm	–	6
Chatzikonstantinou et al. (8)	2020	SBRT	D98%, D90%, V35Gy, V37.5Gy, V42Gy, V52.5Gy	D_max_, V18Gy, V29Gy, V36Gy, V44Gy, D0.1 cm^3^	–	15
**Liver**						
Pennington et al. (9)	2015	SBRT	V100%, V150%, D90%, D_ave_, D_mean_, D_min_	D_mean_, V15Gy	R50%	10
Hass (10)	2019	HDR BT	D90%, D99.9%	V5Gy, RV5Gy	–	85
**Gynecological malignancies**						
Georg (11)	2008	HDR BT	D90%, V7Gy, V3.5Gy	D1ccm, D2ccm, D5ccm, D10ccm, V3Gy, V3.5Gy, V5Gy, V7Gy	–	9
Yildirim (12)	2019	HDR BT	D_min_, D_max_, D_mean_, D2%, D98%, HI, CI	D_mean_, D0.1ccm, D1ccm, D2ccm, D5ccm, D10%, D50%	–	12
**Intrathoracic malignancies**						
Milickovic (13)	2017	SRS	V100%, V95%, V90%, V150%, D95%, D99%, D_min_, CI, COIN	D_max_, D_ave_, D1,000ccm, V_PD_	HTCI, CN, HDS, R50%, D2cm, V20%–V80%	5
**Cerebral malignancies**						
Milickovic (13)	2017	SBRT	V100%, V95%, V90%, V150%, D95%, D99%, D_min_, CI, COIN	D_max_, V_PD_	HTCI, CN, HDS, R50%, D2cm, V20%–V80%	5

PTV, planning target volume; CTV, clinical target volume; OARs, organs at risk; HDR BT, high-dose-rate brachytherapy; SBRT, stereotactic body radiotherapy; SRS, stereotactic radiosurgery; Vx%, volume of a given region receiving x % of the prescribed dose; V_PD_, volume of a given region receiving the prescribed dose; Dxccm, minimal radiation dose for the most irradiated volume of x cm^3^ of a given region; Dx%, minimal radiation dose for the most irradiated volume of x % of a given region; D_min_, minimum dose in a given region; D_max_, maximum dose in a given region; D_ave_, average dose in a given region; RVxGy, relative volume of organ receiving x Gy (in %); HI, homogeneity index; CI, conformity index; HTCI, healthy tissue conformity index; CN, conformation number; COIN, conformal index; HDS, high-dose spillage; R50%, the ratio of the volume receiving 50% of the prescribed dose to PTV; Dxcm, maximum relative dose x cm beyond the PTV in any direction; V20%–V80%, healthy tissue volume (tissue outside the PTV) receiving between 80% and 20% of the prescribed dose.

For prostate, two groups (8, 9) performed dosimetric comparison of real HDR BT plans with virtual SBRT plans, while the third group (10) compared real SBRT plans against virtual HDR BT plans. In general, SBRT revealed significantly better D100% and D99% than HDR BT due to the inherent limitation of HDR BT to fully cover areas inside the PTV that are anteriorly intersecting with the urethra. In terms of dose escalation, HDR BT showed significantly higher average V150% and V200% values compared to SBRT. HDR BT performed significantly better for the dose to the rectum and the bladder but not for the dose inside the urethra in the first (8) and third (10) studies. In the second study (7), HDR BT performed significantly better for rectum but worse for bladder and urethra than SBRT. Both modalities have their advantages and disadvantages regarding the PTV coverage and the sparing of the OARs, with only one dosimetric feature that might be clinically decisive in future clinical trials, namely, the dose escalation inside the prostate PTV, which HDR BT can provide.

In liver malignancies, Pennington et al. (11) dosimetrically compared SBRT and virtual HDR BT, treating the same PTV with both techniques, and concluded that HDR BT could achieve higher target dose, similar dose to OARs, but potentially lower minimum dose to the target. Hass et al. (12) concluded that HDR BT revealed superior outcomes in terms of both target coverage and exposure to the remaining healthy liver.

In gynecologic malignancies, Yildirim et al. (14) used the same PTV to generate plans for both modalities and demonstrated slightly lower but comparable PTV coverage for SBRT plans compared to HDR BT plans and similar doses to the OARs, except the bladder D_2cc_, which was found significantly lower in HDR BT plans. Georg et al. (13) used different PTVs for each modality and attempted to use the high-tech techniques each modality has to offer and revealed inferior performance for SBRT in terms of both target coverage and steepness of dose fall-off. A retrospective survival analysis based on the United States National Cancer Database (17), triggered by an apparent growing clinical implementation of SBRT replacing HDR BT, revealed an apparent increase in mortality risk associated with an increased use of IMRT and SBRT techniques, instead of HDR BT, for delivering the boost dose in cervical cancer patients.

**Figure 1:**
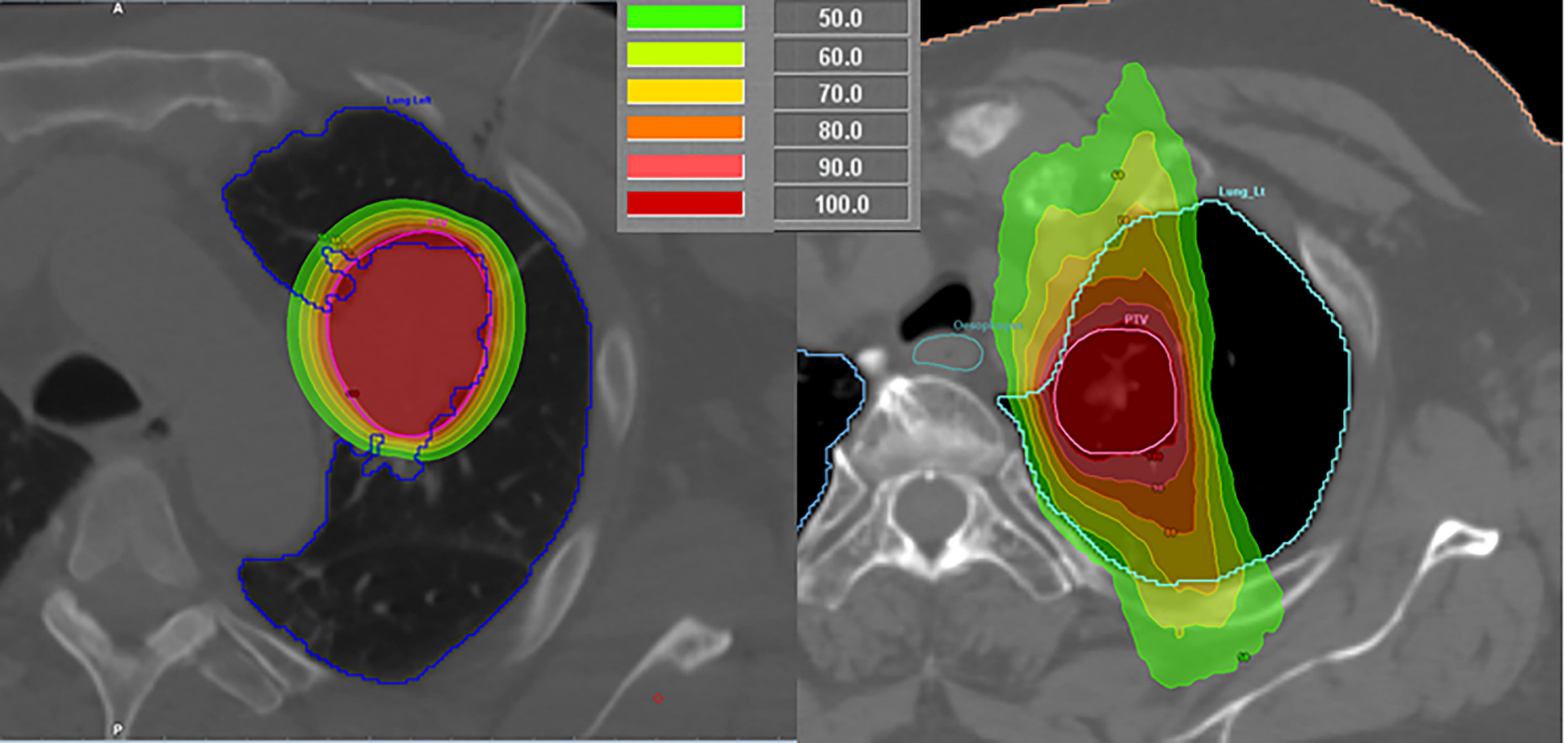
Comparison of a high-dose-rate brachytherapy (left) and stereotactic body radiotherapy (right) plans for lung malignancies in different patients.

For intrathoracic malignancies, SBRT has been shown by Milickovic et al. (15) to be non-superior to HDR BT in its dosimetric aspect. Especially with regard to larger PTVs, HDR BT holds value in covering locations, where SBRT is unable to deliver sufficient dose because of normal tissue dose constraints, because the low-dose regions around the PTV increase much faster for SBRT than in the case of the HDR BT (example in **Figure 1**). The dose fall-off sharpness of the “^192^Ir-knife” is moreover capable of boosting dose inside the PTV with its inherent inhomogeneous dose distribution, leading to a dose escalation that cannot be reached by SBRT for intrathoracic PTVs.

In cerebral malignancies, Milickovic et al. (15) demonstrated that HDR BT can be at least as good as SRS when the anatomical location of the target allows for needle placement. Significant dose escalation with significantly lower intermediate- and low-dose spillage was also demonstrated to be achievable with HDR BT plans.

While the ^192^Ir BT (Iridium-Knife) with its invasive nature requires additional expertise, experience, and logistics in comparison with external beam radiotherapy ablative options (SBRT, SRS), it manifests itself as an extremely “sharp knife” in terms of delivering higher dose gradients and simultaneously reducing the delivered dose to adjacent OARs. Furthermore, the integral dose delivered to the patient can be significantly reduced in comparison to SBRT and SRS treatment options, according to the studies presented herein. Its ability to significantly escalate dose inside the target region may have a key role in the era of personalized treatments, aiding the investigations toward optimal combinations of ablative radiation doses combined with immunotherapy (20) or present a practical solution for tumors with radioresistant hypoxic regions (21). HDR BT and external beam ablative options should be considered equivalent and complementary radiotherapy techniques and chosen based on their merits for each anatomical region and individual patient and tumor characteristics.

## Author Contributions

YR and GA conceived and designed the review, collected and analyzed the representative studies from the literature, and wrote and approved the submitted version.

## Conflict of Interest

The authors declare that the research was conducted in the absence of any commercial or financial relationships that could be construed as a potential conflict of interest.

## Publisher’s Note

All claims expressed in this article are solely those of the authors and do not necessarily represent those of their affiliated organizations, or those of the publisher, the editors and the reviewers. Any product that may be evaluated in this article, or claim that may be made by its manufacturer, is not guaranteed or endorsed by the publisher.
